# The interaction between the PARP10 protein and the NS1 protein of H5N1 AIV and its effect on virus replication

**DOI:** 10.1186/1743-422X-8-546

**Published:** 2011-12-16

**Authors:** Mengbin Yu, Chuanfu Zhang, Yutao Yang, Zhixin Yang, Lixia Zhao, Long Xu, Rong Wang, Xiaowei Zhou, Peitang Huang

**Affiliations:** 1 Institute of Biotechnology, Academy of Military Medical Sciences, Beijing 100071, People's Republic of China; 2 Institute of Chemical Defence, Beijing 102205, People's Republic of China; 3 Institute of Disease Control and Prevention, Chinese Academy of Military Medical Sciences, Beijing, People's Republic of China; 4 Beijing Institute for Neuroscience, Capital Medical University, Beijing 100069, China

## Abstract

**Background:**

During the process that AIV infect hosts, the NS1 protein can act on hosts, change corresponding signal pathways, promote the translation of virus proteins and result in virus replication.

**Results:**

In our study, we found that PARP domain and Glu-rich region of PARP10 interacted with NS1, and the presence of NS1 could induce PARP10 migrate from cytoplasm to nucleus. NS1 high expression could reduce the endogenous PARP10 expression. Cell cycle analysis showed that with inhibited PARP10 expression, NS1 could induce cell arrest in G2-M stage, and the percentage of cells in G2-M stage rise from the previous 10%-45%, consistent with the cell proliferation result. Plague forming unit measurement showed that inhibited PARP10 expression could help virus replication.

**Conclusions:**

In a word, our results showed that NS1 acts on host cells and PARP10 plays a regulating role in virus replication.

## Background

The NS1 protein of avian influenza virus (AIV) is present in host cells infected by the virus instead of being present in mature virions, so it is also called nonstructural protein (NS) [1]. The NS1 protein has two nuclear localization signals, which can induce synthesized NS1 migrate rapidly to nuclei, and aggregate in nuclei early infected by virus. While in late phase of infection, NS1 aggregates in nucleoli and forms a compact crystal-like inclusion body [2].

Studies show that amino-terminal RNA binding region and carboxyl-terminal effector domain of the NS1 protein are closely related to protein synthesis in host cells [3,4]. By binding different types of RNA in host cells, RNA binding region of the NS1 protein can inhibit polyadenylation and splicing of mRNA in host cells, and block protein synthesis [5,6]. Effector domain of the NS1 protein can interact with nuclear protein of host cells, inhibit nuclear export of mRNA, and be used in virus mRNA synthesis [7]. In addition, NS1 can bind dsRNA, inhibit NF-κB activation and IFN-β synthesis, and prevent PKR from activation; NS1 can also inhibit PKR from activation by directly acting on it, and thus inhibit cell apoptosis [8] and make virus exempt from immune reaction in host.

With NS1 of AIV-H5N1 as bait, we screened a protein interacting with NS1 through yeast two-hybrid experiment, i.e. poly (ADP-ribose) polymerases 10 (PARP10), a member of PARP family. Studies showed that all 18 members of PARP family have PARP activity and can modify part of protein in nuclei [9]. Studies also found that the protein family plays certain regulating role in DNA replication and repair [10,11], gene transcriptional regulation [12-14], cell cycle [15], proliferation [16], cell apoptosis and necrosis [17-19]; moreover, PARP family members also play certain modification regulating role in physiological and pathological processes like inflammation [20], tumor [21,22] and aging [23,24].

PARP 10 has many domains. C-terminal PARP domain can modify itself and core histone through PARP activity [16]; Leu-rich nuclear export sequence can promote itself to localize in cytoplasm, and the absence of the sequence can induce PARP10 aggregate in nuclei; 2 C-terminal ubiquitin-binding motifs can regulate nuclear transport of protein [16]. Further study showed that PARP10 can inhibit transformation of rat embryo fibroblasts through interrupting Myc and E1A pathways with its nuclear export sequence [16]. Study also found that during late G1 stage to S stage, PARP10 aggregated in nucleoli participates in regulation of cell proliferation through phosphorylation and binding RNA polymerase I [25].

Synthesized PARP10 in cytoplasm can migrate to nuclei, and this provides a space for interaction between PARP10 and NS1. Therefore, research on their interaction and the physiological function induced can help to explore how PARP10 affects AIV replication. Our study results show that the interaction between PARP10 and NS1 can change cell cycle, and PARP10 can affect virus replication, which provides some clue for the virus replication mechanism in cells.

## Materials and methods

### Cell culture

A549 cells were cultured in McCoy's 5A medium. BHK21, NIH3T3 and MDCK cells were cultured in Dulbecco's modification of Eagle's medium (DMEM). All media were supplemented with 10% fetal bovine serum (Hyclone) and cells were maintained at 37°C in a 5% CO_2 _atmosphere.

### Plasmid construction

cDNA encoding of human PARP10 and NS1 of H5N1 AIV were cloned into pDsRed-C1 and pEGFP-N3 vectors respectively for co-localization experiment. Truncated forms of human PARP10 (as indicated in the figure legends) were generated by PCR and cloned into pCMV-Myc, and cDNA of NS1 were cloned into pCMV-Flag for co-immunoprecipitation. pGEX-6p-1-NS1 was constructed to express the GST-NS1 fusion protein. The DNA sequence corresponding to PARP10 nucleotides 617-635 was subcloned into pEGFP-C1H1U6 vector to transcribe short hairpin RNA (shRNA).

### Antibodies and western blotting

The primary antibodies used were as follows: mouse monoclonal antibodies Anti-β-actin (Promega), anti-Myc (Promega), anti-Flag (Promega), and rabbit anti-PARP10 (Bethyle) were obtained by commercially, and polyclonal antibody anti-M1 was generated by our lab. Horseradish peroxidase (HRP) labeled secondary antibodies were purchased from Santa Cruz Biotech. Western blot analyses of total cell lysate were performed using sodium dodecyl sulfate polyacrylamide gel electrophoresis (SDS-PAGE) methods with 10% polyacrylamide gels. After electrotransfer to polyvinylidene fluoride (PVDF) membranes (Amersham), the interesting proteins were visualized using antibodies as described above.

### Verification of the interaction

For in vitro interaction assays, bacterial expressed GST-NS1 fusion protein was purified through protein purification system ÄKTA^KM ^Purifier. After Myc-PARP10 fusion protein was expressed in A549 cells, whole-cell lysates were prepared in radioimmunoprecipitation assay (RIPA) buffer and centrifuged to obtain supernatant. GST-pulldown was performed as per the instruction of MagneGST™ Glutathione Particle kit (Promega). The GST-NS1 and Myc-PARP10 fusion proteins were identified by Western blotting.

For in vivo interaction assays, A549 cells were transfected with pCMV-Myc-PARP10 and pCMV-Flag-NS1 plasmids for transient expression and whole-cell lysates were prepared in RIPA buffer. Coimmunoprecipitated proteins were detected by Western blot analysis. Myc-PARP10 and Flag-NS1 expression were analysed by Western blotting using whole-cell extracts prepared in RIPA buffer. For immunoprecipitations and Western blot analysis, anti-Flag and anti-Myc antibodies were used. Co-immunoprecipitation was performed as per the instruction of Protein A/G plus-Agarose beads kit (Promega).

### Colocalization analysis

A549 cells were maintained in the center of 35 mm glass Petri dish till 80% confluence, then cotransfected with plasmids encoding NS1 tagged with green fluorescent protein (GFP-NS1) and PARP10 tagged with red fluorescent protein (RFP-NS1) using Lipofectamine 2000 (Ivitrogen) according to the manufacturer's instructions. After 16 h of transfection, cells were rinsed once with pre-cooled phosphate buffered saline (PBS) and added 4% paraformaldehyde to retain cells at 4°C for 5 min. Cells were washed twice with pre-cooled PBS and stained with 500 μl 1 μmol/L 4',6-diamidino-2-phenylindole (DAPI) for 5 min at room temperature. At last, cells were washed three times with PBS, and the co-localization of target proteins was observed under a laser co-focal microscope.

### Cell cycle measured by flow cytometry

The cells transfected in a 6-well plate were digested with trypsin, and centrifuged at 1,000 g for 4 min. The sediment was washed in PBS containing 10% calf serum, and 70% ethanol in PBS was added to fix the cells at -20°C for 4 h. The fixed cells were then washed twice with pre-cooled PBS, incubated for 30 min at 37°C with 1 mg/ml RNaseA solution and stained with 50 μg/ml propidine iodide (PI) for 10 min away from light. The percentages of cells at different stages were measured by flow cytometry.

### Virus proliferation detection

The cells were cultured more than 90% confluence, rinsed twice with Hanks buffer (Gibico), 1 ml serum-free medium and 5 × 10^5 ^pfu H5N1 AIV were added, and then lightly oscillated to mix up. The plate was incubated at 37°C for 1 h, and then cells were rinsed twice with Hanks buffer. 2 ml serum-free medium was added, and samples were cultured at 37°C. Supernatant and infected cells were collected at 12, 24, 36, 48, 60 and 72 h respectively, and supplemented to the same volume with 2 × SDS sample loading buffer. The virus replication was indirectly identified by Western blotting with anti-M1 antibody.

### TCID50 measurement

BHK21 cells transfected with plasmids were cultured for 24 h, and infected with H5N1 AIV. After virus infection for 48 h, the plate was placed overnight at -20°C, then melted, blown and mixed up, and diluted to 10-fold serial dilution. MDCK cells of more than 90% confluence in the 96-well plate were washed twice with Hanks buffer (Gibico), 100 μl serum-free DMEM medium was added to each well, and seeded 4 wells with diluted virus sample. At the same time, wells seeded with H5N1 AIV were used as positive control, and serum-free medium was used as negative control. Cells were cultured in an incubator at 35°C, and the pathological changes were observed every 24 h till no change was found. The observation generally lasted 5-7 d. Virus titer was measured with Reed-Muench method.

## Results

### NS1 interacts with PARP10

**Figure 1:**
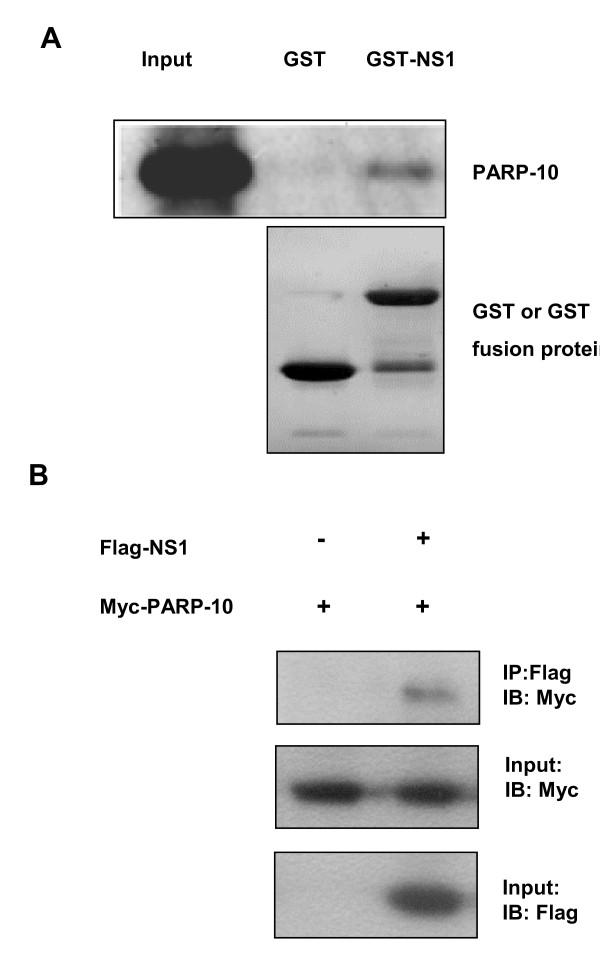
**NS1 could have in vitro and in vivo interaction with PARP10**. **a**. Interaction of GST-NS1 and Myc-PARP10 were identified in GST-pulldown assay. Bacterial expresssed soluble GST and GST-NS1 protein were purified, and SDS-PAGE and Commassie Blue Fast Staining revealed that GST and GST-NS1 of higher purity were obtained. There was a stripe similar to the size of GST under GST-NS1 stripe, indicating that GST-NS1 degraded during the purification. Myc-PARP10 was transient expressed in A549 cells and identified by immuno-blotting. The sediment of GST-pulldown was examined by immno-blotting using anti-Myc antibody. It was found that GST-NS1 could capture Myc-PARP10, while GST could not. **b**. NS1 could have in vivo interaction with PARP10. Myc-PARP10 and Flag-NS1 were transiently expressed in A549 cells, which were lysed in RIPA buffer, co-immunoprecipitated using anti-Myc antibody or anti-Flag antibody, and the sediment obtained was examined by Western blotting. One tenth of each lysate was taken to identify protein expression.

To verify the screening result of yeast two-hybrid system, we identified the in vivo and in vitro interaction between NS1 and PARP10. First, we verified the presence of the interaction with GST-pull down in vitro. At low temperature, induced BL21 had soluble expression of GST-NS1 fusion protein. The protein was about 52 KD, consistent to the size expected. GST and GST-NS1 of high purity were obtained through purification system, and mixed with A549 lysate containing Myc-PARP10 of transient expression, and then used for pull-down assay. The sediment was examined by Western blotting using anti-Myc antibody, and the result showed that GST-NS1 could bind and sediment PARP10, while GST could not (Figure 1a), indicating that NS1 protein in vitro could interact with PARP10. Then, we verified the interaction in vivo between PARP10 and NS1 by co-immunoprecipitation. Myc-PARP10 was expressed individually and co-expressed with Flag-NS1 in A549 cells. After transient expression, the cells were lysed with RIPA lysate, and co-immunoprecipitation was performed. The sediment of co-immunoprecipitation was identified by Western blotting and the result showed that NS1 could interact with PARP10 (Figure 1b).

### C-terminal of PARP10 interacts with NS1

**Figure 2:**
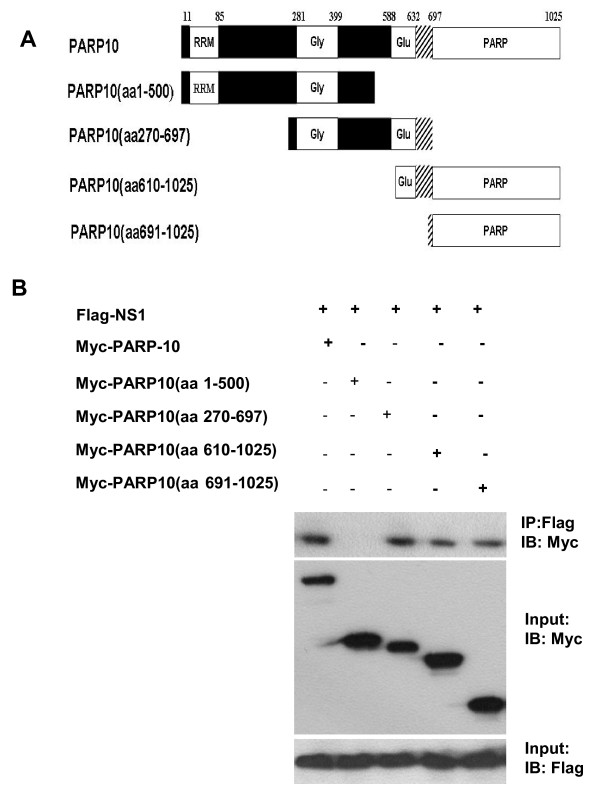
**NS1 interacting C-terminal of PARP10 identified with co-immunoprecipitation**. **a**. The schematic domain architecture of whole/truncated PARP10. PARP10 protein was divided into four fragments according to their corresponding domains: RRM domain and glycine rich region (aa 1-500), glycine rich region and glutamate rich domain (aa 270-697), glutamate rich region and PARP domain (aa610-1025), and PARP domain (aa 691-1025). Nuclear export signal and two ubiquitin-binding motifs are located at the overlapping area of glutamate rich region and PARP domain (aa632-697). **b**. The whole/truncated PARP10 and Flag-NS1 were transiently expressed in A549 cells, which were lysed in RIPA buffer, co-immunoprecipitated using anti-Myc antibody or anti-Flag antibody, and the sediment obtained was examined by Western blotting. One tenth of each lysate was taken to identify protein expression.

PARP10 is made up of 1025 amino-acid residues, and it consists of many domains [16]. To analyze the domains that PARP10 interacts with NS1, PARP10 cDNA was divided into four segments according to its encoded domains (Figure 2a): the first segment was 1503 bp, encoding similar RNA recognition motif (RRM) and glycine rich region; the second was 1287 bp, encoding glycine rich domain and glutamate rich region; the third segment was 1281 bp, encoding glutamate rich domain and PARP domain; and the fourth segment was 1005 bp, encoding PARP domain.

PARP10 was expressed in fragments in A549 cells, and the C-terminal of PARP10 that interact with NS1 was identified with co-immunoprecipitation, i.e. catalytic domain and glutamate rich region of PARP10 (Figure 2b). This also demonstrated that PARP10 and NS1 have physical interaction.

### PARP10 and NS1 can co-localize in nuclei

**Figure 3:**
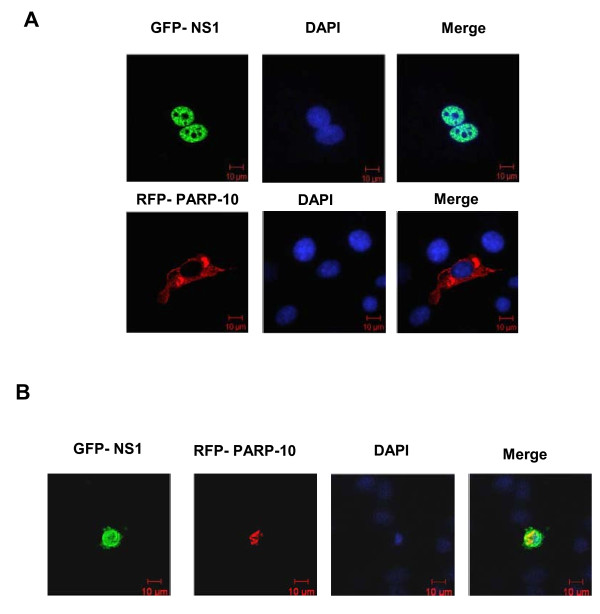
**NS1 co-localized with PARP10 in A549 nucleus**. **a**. GFP-NS1 (green) and RFP-RARP10 (red) were transiently expressed respectively in A549 cells, and nuclei were identified by DAPI (blue) staining. Observation under the microscope showed that GFP-NS1 was mainly localized in nuclei, while RFP-PARP10 was mainly localized in cytoplasm. **b**. When the two were co-expressed in A549 cells, RFP-PARP10 migrated from cytoplasm to nuclei and overlapped with the florescent shed by GFP-NS1, indicating that presence of NS1 could induce localization change of PARP10.

Localization of PARP10 and NS1 could be directly observed from cell-level expression of RFP-PARP10 and GFP-NS1 in A549 cells. Results showed that when PARP10 fused with red fluorescent protein was transiently expressed in A549 cells, it was localized in cytoplasm, while NS1 with green fluorescent label was localized in nuclei (Figure 3a). If the two proteins were transiently co-expressed in A549 cells, then the localization of PARP10 would change and mainly aggregate in nuclei, and the red fluorescent could overlap with green florescent, indicating that NS1 could change the localization of PARP10 (Figure 3b). The localization result in NIH3T3 cells was same to that in A549 cells. So the results illustrated that NS1 could interact with PARP10 and effect PARP10's location in cells.

### NS1 inhibits PARP10 expression

**Figure 4:**
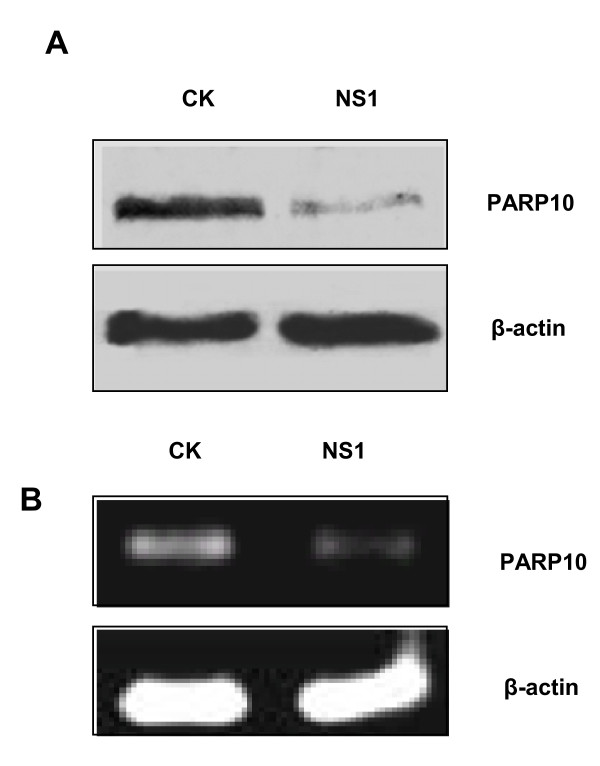
**NS1 of high expression in A549 cells could reduce endogenous PARP10 expression**. **a**. NS1 of H5N1 AIV was transiently expressed in A549 cells, which were then lysed with RIPA buffer, and examined by Western blotting using anti-PARP10 monoclonal antibody, with β-actin as internal control. It was found that NS1 could reduce PARP10 expression level. **b**. RT-PCR assay found that with GAPDH as internal control, PARP10 saw a low RNA expression level because of the high expression of NS1.

NS1 protein molecules can inhibit protein synthesis and increase virus protein replication by interrupting normal mRNA splicing and nuclear export [3-6]. As a kind of host protein, was PARP10 affected by NS1? We made high expression of NS1 in cells and Western blot analysis showed that endogenous PARP10 expression level decreased (Figure 4a); RT-PCR assay also found that NS1 could reduce the transcription of endogenous PARP10 (Figure 4b). In a word, NS1 can inhibit PARP10 expression.

### Expression magnitude of PARP10 and NS1 could change cell cycle

**Figure 5:**
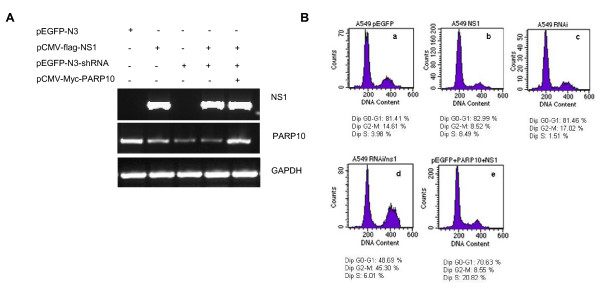
**PARP10/NS1 expression level could change cell cycle of A549**. **a**. Total RNA was extracted from transfected cells, and NS1 and PARP10 transcription level were identified by RT-PCR. PARP10 siRNA could significantly reduce PARP10 transcription level; NS1 transient expression had less inhibition on PARP10, while PARP10 expression vector could effectively express the target proteins. **b**. Transfect cells was analyzed by flow cytometry, and it was found that when NS1 transient expression and PARP10 knock-down were performed together, the percentage of A549 cells in G2-M stage grew to 45% from 10%. When PARP10 expression level was elevated, the percentage of cells in G2-M stage saw significant decrease, similar to the percentage of cells transfected with empty vector, but the percentage of cells in G1-S stage grew from less than 10% to 20%.

Over expression or lowered expression of PARP10 can affect cell cycle [25]. We investigated the effect of NS1 and PARP10 on cell cycle by flow cytometry through regulating NS1 and PARP10 expression level. First, PARP10 and NS1 expression in each sample were examined respectively by RT-PCR. Results showed that NS1 and PARP10 expression vector could effectively express the target proteins, and small interfering RNA (siRNA) designed for PARP10 coding sequence could effectively inhibit PARP10 expression (Figure 5a). Flow cytometry analysis found that with PARP10 expression inhibition, NS1 could induce cell arrest in G2-M stage. When PARP10 expression rebounded, the effect of the NS1 protein on cell cycle change disappeared almost (Figure 5b), indicating that NS1 and PARP10 expression level could change the cell cycle of A549.

### PARP10 can inhibit the proliferation of H5N1 AIV in cells

**Figure 6:**
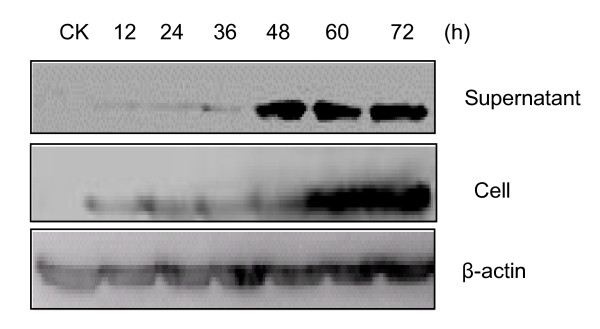
**Proliferative kinetics of AIV H5N1 in BHK21 cells**. BHK21 cells were infected directly with H5N1 AIV, and the supernatant and bottom cells of the sample at 12, 24, 36, 48, 60, 72 h were collected respectively. Finally, samples separated were examined by Western blotting with anti-M1 antibody respectively, with β-actin as internal control. The result showed that the volume of the M1 protein had significant growth at 48 h, and less obvious growth afterward.

The M1 protein is a structural protein of avian influenza virus and the virus level can be detected indirectly through Western blotting of the M1 protein. We used H5N1 AIV to infect A549, COS7 and BHK21 cells, respectively. The Virus replication magnitude had significant increase in the supernatant of BHK21 cells 48 h after the infection, had significant increase in the cells 60 h after the infection, and no significant increase in the supernatant and in the cells afterwards, indicating that H5N1 AIV replication reached the peak in BHK21 cells at 48 h (Figure 6). Therefore, we chose BHK21 cells and 48 h after infection to investigate the effect of PARP10 on AIV proliferation.

**Figure 7:**
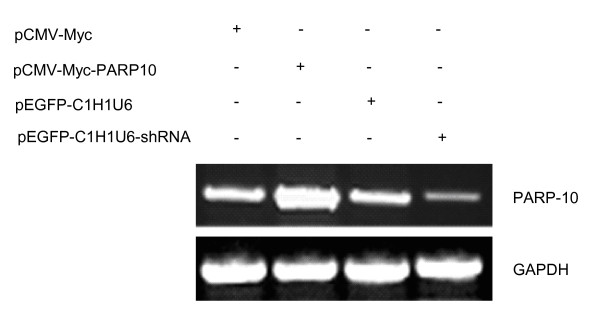
**PARP10 expression level measured with RT-PCR**. With GAPDH as internal control, RT-PCR found that PARP10 expression plasmids in BHK21 cells could effectively express target gene, while PARP10 siRNA transcription plasmids could effectively inhibit target gene expression.

PARP10 expression plasmids and PARP10 siRNA transcription plasmids were transfected into BHK21 cells respectively, with corresponding empty vector as control, and PARP10 expression in each sample was detected. It was found that PARP10 expression plasmids could effectively express PARP10, while PARP10 siRNA transcription plasmids could effectively inhibit PARP10 expression (Figure 7). Another group of samples transfected at the same time was infected with H5N1 AIV, and the virus were collected 48 h after the infection to infect MDCK cells. Plaque forming unit (PFU) of each virus sample was computed using TCID_50 _when multiplicity of infection (MOI) was diluted to 2, 0.2, 0.02 and 0.002_. _The data was summarized in Table 1 and 2, which were one-way ordinal 4 × 2 contingency tables. The data was analyzed with rank sum test, and the result showed *P *= 0.0001 (*P *< 0.01), indicating the difference was of statistical significance. In this experiment, other different MOI could also back this result.

**Table 1:** The effect of the PARP10 protein high expression on the virus replication <

MOI	2	0.2	0.02	0.002
Control group(a)	100 ± 0.00	50 ± 2.50	< 8.3 ± 0.00	< 8.3 ± 0.00

Experiment group(b)	62.5 ± 4.33	< 8.3 ± 0.00	< 8.3 ± 0.00	< 8.3 ± 0.00

Note: Data (mean ± SD, n = 4) in the table is the percentage of MDCK cytopathy caused by different H5N1 AIV dilution which is the value of cytopathy cells/total cells; *P *< 0.01. TCID_50 _was obtained from the data and PFU was converted from TCID_50 _with formula logTCID_50 _= log pfu + 0.67. The pfu of the two samples was as the follows: pfu (a) = 10^-4.17^/ml, pfu (b) = 10^-3.87^/ml

**Table 2:** The effect of the PARP10 protein expression inhibition on the virus replication

MOI	2	0.2	0.02	0.002
Control group(c)	70 ± 4.33	18.3 ± 2.89	< 8.3 ± 0.00	< 8.3 ± 0.00

Experiment group(d)	100 ± 0.00	70 ± 5.00	18.3 ± 1.44	< 8.3 ± 0.00

Note: Data (mean ± SD, n = 4) in the table is the percentage of MDCK cytopathy caused by different H5N1 AIV dilution which is the value of cytopathy cells/total cells; *P *< 0.01. TCID_50 _was obtained from the data and PFU was converted from TCID_50 _with formula logTCID_50 _= log pfu + 0.67. The pfu of the two samples was as the follows: pfu (c) = 10^-4.06^/ml, pfu (d) = 10^-5.06^/ml

The result showed that H5N1 AIV magnitude decreased in case of PARP10 transient expression in BHK21 cells, and H5N1 AIV magnitude grew in case of PARP10 knock-down in BHK21 cells.

## Discussion

We first verified the interaction between NS1 and PARP10 with co-localization, co-immunoprecipitation and GST-pull down. Cell co-localization found that the presence of NS1 could induce the PARP10 protein localized in cytoplasm to migrate from cytoplasm to nuclei, indicating that NS1 could change localization and function of PARP10. As PARP10 mainly localized in nuclei under the action of nuclear export inhibitor, we supposed that NS1 might inhibit the nuclear export of PARP10 in nuclei, and make it remain in the nuclei. Further study found that NS1 acts on Glu-rich region and PARP domain of PARP10, and Glu-rich region contains potential nuclear export signal and two ubiquitin interaction motifs (UIM). Some studies report that UIM play certain regulating role in nuclear export and import in some proteins [26-28]. The interaction between NS1 and PARP10 might block nuclear export signal (NES) and UIM of PARP10. As NS1 has two nuclear export signals, NS1 and PARP10 are co-localized in nuclei under the action of nuclear export signal of NS1. NS1's action on catalytic domain of PARP10 may affect the enzymatic activity of PARP10. It is reported that NS1 can promote virus replication through interacting with many proteins of the host and interrupting the normal expression regulation of host cells. Expression profiles of human and mouse tissues show that PARP10 is a widely expressed protein [16], indicating that PARP10 has wide and fundamental biological functions, and may play certain role in some basic pathways. Therefore, the interaction between NS1 and PARP10 may involve some basic biological functions of cells, and also involve some general protein molecules in signal transduction and protein expression regulation.

Individual NS1 protein expression and PARP10 knock-down did not have significant effect on cell cycle in A549 cells, but the NS1 protein expression and PARP10 knock-down together would significantly induce cell arrest in G2-M stage, with percentage of cells in G2-M stage increased from the previous 10%-45%, consistent to the cell proliferation result. When PARP10 siRNA transcription plasmids, NS1 expression plasmids and PARP10 expression plasmids were co-transfected, it was found that the percentage of cells in G2-M stage saw significant decrease, back to the percentage of cells transfected by empty vector, but the percentage of cells in G1-S stage grew from less than 10%-20%, indicating that co-transfection promoted cells progress into S stage. However, there was a contradictory result that the percentage of cells in G2-M stage when NS1 protein expression only was not above the percentage of empty vector, this may be due to the PARP10 expression level. When PARP10 expression was inhibited significantly, the cells would be apt to G2-M stage. When PARP10 expression was inhibited slightly, the cells would be not apt to G2-M stage. Therefore, this also indicated that NS1 protein of AIV interacted with various proteins to change cell cycle and facilitate AIV infection.

AIV could have quick proliferation in MDCK cells and induce significant pathological changes, but MDCK cells have a low transfection rate, and are not suitable for this study. As AIV is quite selective for hosts, to better detect PARP10's effect on virus replication, we explored the proliferation of H5N1 AIV in A549, BHK21 and COS7 cells. It was found that the virus replication had significant growth in BHK21 cells, but slower proliferation in the other two. As AIV had effective replication in BHK21 cells and the log growth period of the virus was between 36 h and 48 h, BHK21 cells were used as host cells of AIV.

After host cells were decided, we explored the effect of BHK21 cells on virus proliferation through PARP10 over expression or knock-down, with 48 h after the infection as starting point of the detection. The analysis of PFU showed that PARP10 over expression induced virus replication decrease, while PARP10 expression inhibition induced virus replication growth, indicating that AIV replication is regulated by PARP10 protein molecule, and PARP10 expression inhibition can promote virus replication.

In summary, PARP10 can interact with NS1, and the interaction can affect cell cycle and virus replication. NS1 might inhibit activity of host cells and promote virus proliferation through the interaction with PARP10. The findings provide clue and foundation for virus replication mechanism in cells.

## Conclusions

NS1 of AIV is expressed early in hosts and interacts with PARP10 to interfere with cell cycle and promote virus replication. This work is helpful to understand the mechanism of AIV infection and further work is required to explore the process of virus replication in the cells.

## List of abbreviations

AIV: Avian influenza virus; NS: Nonstructural protein; PARP10: Poly (ADP-ribose) polymerases 10; DMEM: Dulbecco's modification of Eagle's medium; shRNA: Short hairpin RNA; HRP: Horseradish peroxidase; SDS-PAGE: Sodium dodecyl sulfate polyacrylamide gel electrophoresis; PVDF: Polyvinylidene fluoride; RIPA: Radioimmunoprecipitation assay; GFP-NS1: Green fluorescent protein; RFP-NS1: Red fluorescent protein; PBS: Phosphate buffered saline; DAPI: 4',6-diamidino-2-phenylindole; PI: Propidine iodide; RRM: RNA recognition motif; siRNA: Small interfering RNA; PFU: Plaque forming unit; MOI: Multiplicity of infection; UIM: Ubiquitin interaction motifs; NES: Nuclear export signal.

## Competing interests

The authors declare that they have no competing interests.

## Authors' contributions

MBY, CFZ, YTY mainly carried out construction of expression plasmids, GST-pulldown, co-immunoprecitation assays, and wrote the manuscript. ZXY and LX contributed to viruses' culture. LXZ and RW contributed to electron microscopic analysis. XWZ contributed to flow cytometric analysis. XWZ and PTH conceived the studies and participated in experimental design and coordination. All authors read and approved the final manuscript.
